# Mineralogical and Technological Characterization of Zeolites from Basin and Range as Pozzolanic Addition of Cement

**DOI:** 10.3390/ma15072684

**Published:** 2022-04-06

**Authors:** Giovanna Montesano, Piergiulio Cappelletti, Domenico Caputo, Barbara Liguori, Assunta Campanile, Concetta Rispoli

**Affiliations:** 1 Dipartimento di Scienze della Terra, dell’Ambiente e delle Risorse, University of Naples Federico II, Complesso Universitario Monte Sant’Angelo, ED. 10, Via Cintia 26, 80126 Naples, Italy; giovanna.montesano2@unina.it (G.M.); piergiulio.cappelletti@unina.it (P.C.); concetta.rispoli@unina.it (C.R.); 2 Applied Chemistry Labs-Department of Chemical, Materials and Industrial Engineering, University of Naples Federico II, 80125 Naples, Italy; domenico.caputo@unina.it (D.C.); assunta.campanile@unina.it (A.C.)

**Keywords:** zeolites, blended cement, pozzolanic addition

## Abstract

The present paper assesses petrographic, mineralogical, chemical, and technological features of different zeolitic tuff samples from various western USA districts of the Basin and Range Province containing mainly erionite, mordenite, clinoptilolite/heulandite and phillipsite. The aim of this characterization is to evaluate the pozzolanic activity of these samples according to European normative UNI-EN 196/5 (Fratini test) to program a possible use as addition for blended cements. Petrographic and mineralogical results show that the two phillipsite-bearing tuffs have a higher theoretical Cation Exchange Capacity (CEC) than the other samples; technological characterization shows a pozzolanic behavior for all the samples but higher for the tuff samples containing phillipsite, which shows a higher reactivity with CaO. All the samples could be thus advantageously employed for the preparation of blended cements, potentially reducing CO_2_ emissions by 70–90%.

## 1. Introduction

Cement is a key building material for worldwide infrastructures, but its production is strictly linked to the CO_2_ emissions. Since about 10% of the global CO_2_ derives from the cement production [1,2], it is necessary to limit the emission of this gas using supplementary cementitious materials (SCM), such as cement, but requiring a smaller amount of energy for their production. The most suitable SCM is certainly natural pozzolan.

Pozzolan *s.s.* is a volcanic incoherent to pseudo-coherent pyroclastic deposit linked to the Neapolitan Yellow Tuff formation [3] with grain size varying from fine to coarse ash and with subordinated lapilli (pumices and scoriae). It has a typical light grey color and comprises very fine pumiceous and vitreous fragments as well as fine ash, both with an alkali-trachytic composition [4,5,6]. Nowadays, the name pozzolan suits any kind of finely grained, mainly glassy volcanic ash [5] but, as the name suggests, pozzolan *s.s*. was mined in the area of Pozzuoli (Naples, southern Italy).

The *pulvis puteolana*, mentioned as “Cuma’s sand” in the Vitruvius’ *De Architectura*, is the most important Phlegraean product as it has been used since the Roman epoch in the production of long-lived cements with a quick setting underwater [7].

Since the late Renaissance, pozzolan has been and is nowadays used for the preparation of hydraulic cements, thanks to properties (e.g., the lime setting and the water resistance) deriving from its composition. As a matter of fact, pozzolan is mainly made of poorly crystalline to completely amorphous silica (SiO_2_) and alumina (Al_2_O_3_), which react quickly (because of the large surface area and non-crystalline nature of their grains and their siliceous composition) with the lime and water, forming calcium hydrate silicates (C-S-H) and calcium hydrate aluminates (C-A-H). Upon hardening, these hydrated Ca-Al silicates display high-mechanical resistance either in aerial or in sub-aqueous environments [7].

The shortage of natural pozzolans has led to the search for materials displaying pozzolanic activity (PAM), i.e., the capability to easily react with lime. There are many siliceous materials which behave as pozzolans: blast furnace slags (BFS), fly ash (a secondary product of coal combustion) and silica fume (a by-product of the silicon industry) but great attention has been paid recently to zeolitic tuffs [8].

The use of zeolitic tuffs as pozzolanic additions for the cement production is an increasingly widespread practice, given the unavailability of natural pozzolan in several countries (e.g., Bulgaria, Germany, Turkey, China, Russia, and USA) and, conversely, the considerable abundance in these countries of zeolites [2,9].

The use of zeolitic tuffs to produce pozzolanic cements is of great industrial interest because zeolite-bearing cements have properties comparable with respect to those made with natural pozzolans. In fact, zeolitic tuffs can even display a better pozzolanic behavior than the pozzolan itself [10].

Moreover, pozzolan has a lower cost than clinker, making pozzolanic cement cheaper with respect to common Portland cements, and above all, with its use it is possible to considerably reduce the consumption of the fuel [2,11,12] necessary for the preparation of the clinker, thus reducing CO_2_ emissions. This, together with the excellent technological properties of pozzolanic cement, makes its use extremely advantageous both economically and environmentally, and its study is of great interest nowadays.

In this paper, mineralogical and technological features of some tuff samples from Basin and Range Province were considered to shed some light on the correlation between the presence of different zeolitic types, theoretical Cation Exchange Capacity (CEC) and pozzolanic behavior. This research represents an attempt to characterize these tuffs as possible re-usable material, defining their technological characteristics for possible planning of future applications.

## 2. Geological Setting, Samples Localities and General Remarks

**Figure 1:**
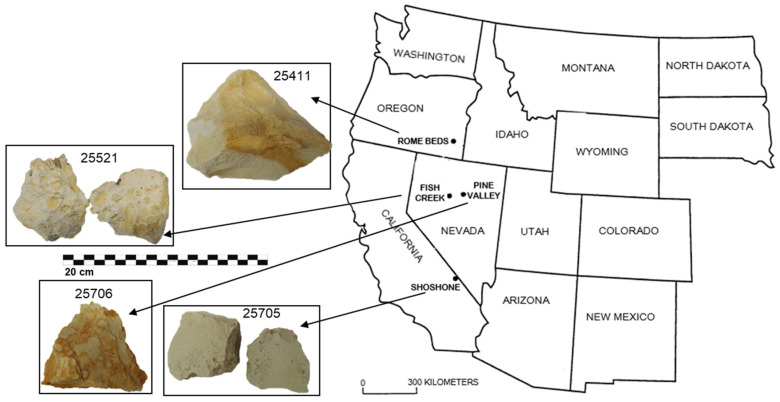
Basin and Range Province sketch map with samples localities (modified after [18]).

Zeolites in deposits of saline, alkaline lakes (fluids with pH = 9.5–10) are widespread, and these settings contain the largest relatively pure concentrations of natural zeolites [10]. Phillipsite, clinoptilolite erionite, and the less common mordenite and chabazite easily form from interaction between fluids of saline, alkaline lakes and silicic vitric ash. The deposits of the western United States in Miocene to Pleistocene Lake (e.g., Teels Marsh in Nevada, Big Sandy Formation in Arizona, Green River Formation of Utah, and Lake Tecopa deposits of California; see Refs. [13,14,15,16]), contain beds of clinoptilolite, phillipsite, erionite, chabazite and analcime. It has been proved that their formation from silicic glass is mainly determined by pore-fluid composition. Among the abovementioned western United States provinces is the Basin and Range (Figure 1). It is an exceedingly complex area of the western Cordillera resulting from the superposition of 600 million years of recurring tectonic and volcanic activity. In the late Cenozoic, the already highly fragmented geology of the Basin and Range was further disrupted by subsidence, by the covering of approximately half the province by alluvial deposits in the valleys, and by the exposure of different stratigraphic and structural levels in adjacent ranges [17].

### 2.1. Rome Beds, Oregon

The Rome area is a northern extension of the Basin and Range Province. Intermittent gentle folding affected the area from Miocene to Pleistocene, and Pliocene folding resulted in the development of local basins which received sediments, including the Rome basin [19]. The Pliocene Rome Beds (Figure 1) are composed of tuffaceous mudstones, volcanic and lithic (epiclastic) sandstones and conglomerates of lacustrine and fluvial origin. Diagenesis produced different types of zeolites, phyllosilicates, carbonates, iron oxides, gypsum and other authigenic minerals. Erionite-bearing rocks (mostly lacustrine tuffs) are frequent in the Miocene Rome Beds, over an elongated north–south area between Rome and Crooked Creek to the west [20].

### 2.2. Fish Creek, Nevada

The Fish Creek Mountains in central Nevada (Figure 1) form a circular-shaped range that covers an area of about 518 km^2^ [21]. Fish Creek Mountains has a broad arcuate southern edge. The profile of the range shows a rim of uniform height (about 460 m) above the surrounding valley floor and a broad shallow central basin (several hundred feet lower than the rim; [21]. The southern and western part of the range form the highest and widest part of the rim, and in this region Fish Creek Mountains were eroded into very rugged badland topography that is associated with a dendritic drainage pattern. The Fish Creek Mountains are composed almost entirely of a single ash-flow deposit, the Fish Creek Mountains Tuff, a crystal-rich rhyolitic tuff erupted by an early Miocene volcano. The eruption was probably of a low-energy type and formed a relatively thick pile of tuff. This tuff shows little lithologic variation [21].

### 2.3. Shoshone, California (Sample 25705)

Composed primarily of Cenozoic rocks [22,23,24], the Shoshone terrane (Figure 1) is located within the Black Mountains block of the Death Valley region (eastern California) and covers an area of about 1300 km^2^ [25]. Volcanic rocks of this terrane consist predominantly of rhyolites but also include dacites, andesites and basalts. The so-called “Shoshone Volcanics” are included within the rhyolitic rocks as an accumulation of lava flows and tuffs, as much as 900 m thick. They consist of pumice lapilli tuff and welded tuff: the welded units contain abundant plagioclase, hornblende, and biotite phenocrysts, whereas the unwelded units consist of blocks of andesite and rhyolite, rare clasts of dolomite and quartzite, and a devitrified pumice lapilli matrix [26]. Clastic sediments deriving from the igneous rocks (pumices), whose vesicles are locally filled with zeolites, were deposited in local basins [27].

### 2.4. Pine Valley, Nevada (Sample 25706)

Pine Valley basin (Figure 1) is an asymmetrically actively subsiding half graben situated in northeastern Eureka County, Nevada. Unlike most valleys in the Basin and Range province, Pine Valley is significantly dissected, and the dissection began after this formerly closed basin was captured by Pine Creek River. The filling of Pine Valley is known as the Hay Ranch Formation, whose exposed rocks are mainly Pliocene and Pleistocene in age according to paleontological evidence [28,29]. This formation is composed of tuffaceous siltstones, sandstones, conglomerates, limestones, and mudstones; these last containing thin volcanic ash beds altered to zeolites [30] and overlies the older Miocene volcanic and sedimentary rocks of the Humboldt Formation [31].

When the Hay Ranch Formation was deposited, the Pine Valley basin drained internally. Lacustrine deposits found along the basin axis, along with paleocurrent indicators and the slopes of Pleistocene terraces, indicate that the basin was closed [32]. Pine Valley was opened by Pine Creek river sometime after deposition of 0.6 myr-old ash layer [31].

## 3. Materials and Methods

In this work, petrographic, mineralogical and chemical analyses were performed on the four tuff samples from Rome Beds (25411), Fish Creek Mountains (25521), Shoshone (25705) and Pine Valley (25706) (Figure 1) at the Department of Earth, Environmental and Resources Sciences (DiSTAR) of the University of Naples Federico II, whereas technological tests were mainly devoted to establishing the pozzolanic activity of the investigated materials were conducted at the Department of Chemical, Materials and Industrial Production Engineering (DiCMAPI) of the University of Naples Federico II.

Petrographic analysis was carried out on thin sections through polarized optical microscopy (POM) with a Leica Laborlux 12 Pol microscope (Leica Camera, Wetzlar, Germany).

Qualitative and quantitative phase analysis was performed by means of X-ray powder diffraction (XRPD and QXRPD, respectively) using a Malvern Panalytical X’Pert Pro diffractometer equipped with a RTMS X’Celerator and a X’Pert High Score Plus 3.0c software (Malvern PANalytical, Almelo, The Netherlands).

Operating conditions were: CuKα radiation, 40 kV, 40 mA, 2θ range from 4 to 70°, equivalent step size 0.017°2θ, equivalent counting time 120 s per step. Data sets were analyzed using RIR/Rietveld method [33,34] with internal standard and TOPAS 5 software (BRUKER AXS Company). Powders with grain size <10 µm were obtained using a McCrone micronizing mill (agate cylinders and wet grinding time of 15 min; Retsch-Alle, Haan, Germany). An α-Al_2_O_3_ internal standard (1 µm, Buehler Micropolish) was added to each sample at a rate of 20 wt.%.

Starting atomic coordinates for identified phases were taken from literature [35] and were the following: erionite [36], mordenite [37], clinoptilolite [38], heulandite [39], calcite [40], phillipsite [41], searlesite [42], quartz [43], mica [44], K-fekdspar [45], and plagioclase [46]. Background profile was fitted using a Chebyshev polynomial function with variable number of coefficients (5–12); diffraction peak profiles were modeled refining crystallite size and strain (Lorentzian contribution) coefficients and two Gaussian coefficients. Unit cell parameters along with weight fractions were also refined. PO (preferred orientation) was treated with March–Dollase approach [47], whenever needed. All agreement index R*_wp_* are below 8.

X-ray fluorescence spectroscopy (XRF; AXIOS Panalytical Instrument; Malvern PANalytical, Almelo, Netherlands) have been performed to determine the chemical composition of samples in the form of pressed pellets. The oxides of the major elements determined were SiO_2_, TiO_2_, Al_2_O_3_, Fe_2_O_3_tot, MnO, MgO, CaO, Na_2_O, K_2_O and P_2_O_5_, whose concentrations are expressed in weight percentages (wt.%). The trace elements determined were Rb, Sr, Y, Zr, Nb, Ba, Cr, Ni, Sc and V, whose concentrations are expressed in ppm (parts per million). Accuracy and precision are generally of 1–2% for the major elements and 5–10% for the trace elements [48].

The weight loss on ignition (LOI) was determined with standard thermo-gravimetric techniques, by predrying 1 g of powder of the sample overnight at 110 °C and then heating the sample to 1000 °C for 4 h.

Micro-textural observations and quantitative micro-chemical analyses were carried out by Scanning Electron Microscopy coupled with Energy Dispersive Spectroscopy (SEM/EDS; Zeiss Merlin VP Compact and JEOL JSM-5310 coupled with Oxford Instruments Microanalysis Unit equipped with an INCA X-act detector; Carl-Zeiss-Strasse, Oberkochen Germany and Jeol Ltd., Tokyo, Japan respectively). Measurements were performed with an INCA X-stream pulse processor (using a 15-kVprimary beam voltage, 50–100 A filament current, variable spot size, from 30,000 to 200,000× magnification, 20 mm WD and 50 s net acquisition real time). The INCA Energy software was employed using the XPP matrix correction scheme and the pulse pile up correction. The quant optimization was carried out using cobalt (FWHM–full width at half maximum peak height- of the strobed zero = 60–65 eV). The following Smithsonian Institute and MAC (Micro-Analysis Consultants Ltd., Saint Ives. UK) standards were used for calibration: diopside (Ca), fayalite (Fe), San Carlos olivine (Mg), anorthoclase (Na, Al, Si), rutile (Ti), serandite (Mn), microcline (K), apatite (P), fluorite (F), pyrite (S) and sodium chloride (Cl). Precision and accuracy of EDS analyses are reported in [49].

The chemical method used to evaluate pozzolanic activity is the Fratini’s test, still accepted as European Standard (UNI EN 196-5, [50]) despite dating back to more than fifty years ago [51,52]. This test allowed us to estimate the amount of Ca(OH)_2_ leached from 20 g of blended cement mixed with 100 mL of deionized water and kept at 40 °C for 8 days. At the end of the experiment, Ca^2+^ and OH^−^ concentrations in the contact solution are estimated using volumetric analysis methods (i.e., complexometric titration with ethylenediaminetetraacetic acid-EDTA and acid-base titration, respectively). Experimental results (i.e., the average values of runs performed in triplicate) have been reported in a plot of Ca(OH)_2_ (expressed as CaO) solubility at 40 °C as a function of OH^-^ concentrations in solution (i.e., vs. alkalinity). Points representing under-saturated solutions, and thus proving the existence of pozzolanic activity given by the specific mineral addition to the OPC, should be under the curve, which means that some of the lime resulting from the hydrolysis of the clinker was fixed by the pozzolanic materials. On the contrary, points above and on the curve represent over-saturated and saturated solutions, respectively, indicating the lack of pozzolanic activity.

## 4. Results and Discussion

### 4.1. Optical Microscopy, XRPD Analysis and SEM Observations

All the four samples show the most common features of zeolitic tuffs, with presence of both pyrogenic and secondary mineralogical phases.

**Figure 2:**
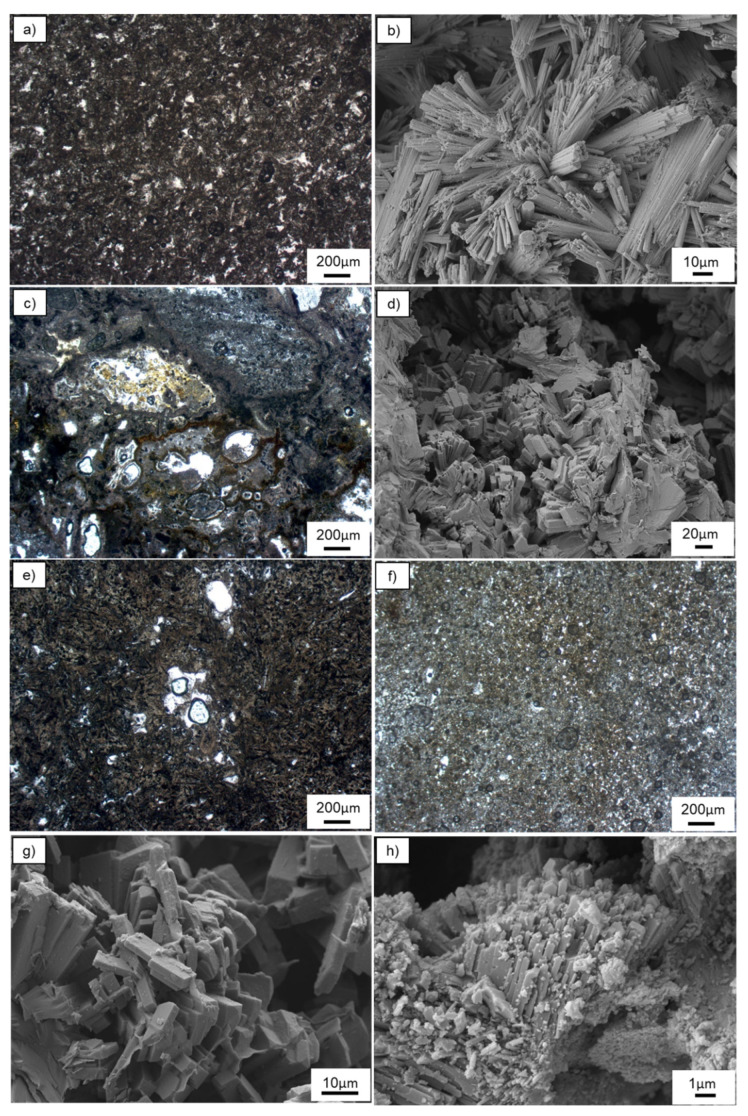
Crossed polarized light microphotograph (**a**) and SEM (**b**) images of sample 25411; crossed polarized light microphotograph (**c**) and SEM (**d**) images of sample 25521; crossed polarized light microphotographs of (**e**) 25705 and (**f**) 25706 samples; SEM images of sample (**g**) 25705 and (**h**) 25706 samples.

The 25411 sample (Figure 2a) shows a reddish color and is microcrystalline, consisting of erionite and mordenite crystals with dimension ranging from microns to sub-microns set into a cineritic matrix. At SEM (Figure 2b), erionite crystals are homogenously distributed in the whole sample and appear as bundles. Occasionally, individual erionite fibers with a clearly hexagonal shape have been observed.

The 25521 sample (Figure 2c) has a cineritic matrix with abundant pumices. The micrometric to sub-micrometric subhedral white clinoptilolite crystals with prismatic-lamellar habit are commonly dispersed in both the matrix and in the pumice voids. Clinoptilolite crystals appear at SEM (Figure 2d) as lamellar, forming layered or sheeted structures. Moreover, the presence of aggregates of acicular/fibrous mordenite crystals has been occasionally noticed.

The 25705 sample (Figure 2e) shows a reddish color and is microcrystalline, with prismatic phillipsite crystals and subordinate opaque minerals set into a cineritic matrix.

The 25706 sample (Figure 2f) has a grayish-white color and a microcrystalline texture, consisting of prismatic phillipsite crystals and opaque minerals set into a cineritic matrix.

Scanning electron microscopy revealed that phillipsite crystals of the 25705 (Figure 2g) sample appear prismatic, sometimes slightly elongated and with a stocky habit, whereas phillipsite crystals from the 25706 sample (Figure 2h) are arranged in aggregates and display less regular and defined shapes with respect to the 25705 sample.

**Figure 3:**
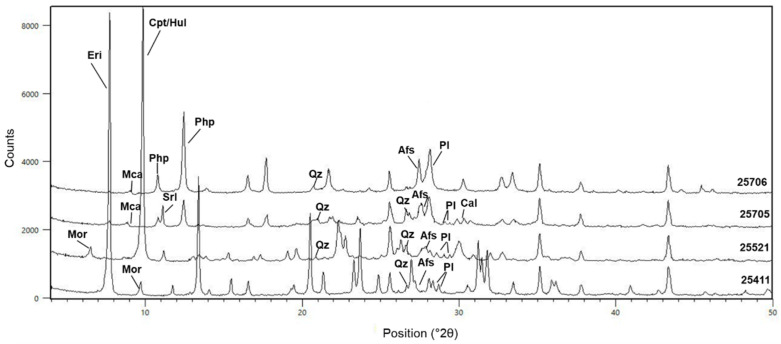
XRPD patterns of the investigated tuff samples. Abbreviations: Eri = erionite; Mor = mordenite; Pl = plagioclase; Hul = heulandite; Cpt = clinoptilolite; Php = phillipsite; Cal = calcite; Mca = mica; Afs: alkali feldspar; Qz = quartz. Mineral abbreviations from [53].

Quantitative XRPD analysis has been carried out to give the amount of the detected mineralogical phases (Table 1). XPRD patterns are shown in Figure 3.

According to quantitative XRPD analysis (Table 1), amorphous content lies between 8 and 15 wt.% for the 25411 and 25521 samples, respectively, and is significantly higher for the 25705 and 25706 samples, reaching about 51 wt.% for the former and 25 wt.% for the latter. This percentages comprise the amount of clay minerals since they are low-order mineralogical phases.

The 25411 sample contains mainly erionite (79 wt.%), and the 25521 sample contains mainly clinoptilolite/heulandite (51 wt.%), with not negligible amounts of mordenite (19 wt.%) and subordinate quartz, alkali feldspar and calcite. The 25705 and 25706 phillipsite-bearing tuffs are quite different, showing different amorphous content (26 and 25, respectively) and different amounts of phillipsite (25 and 64 wt.%, respectively). Both the samples contain subordinate alkali feldspar and quartz, only the 25705 sample also contains plagioclase, erionite, calcite and searlesite, whereas only the 25706 sample contains mica as subordinate mineralogical phase.

These investigations, especially QXRPD analysis, allowed us to establish that all samples (except for 25705) can be considered as “zeolitites”, containing almost completely zeolites.

### 4.2. Whole-Rocks Compositions and Zeolite Crystal Chemistry

Major element analyses and loss on ignition (LOI) values of the investigated samples are reported in Table 2.

**Figure 4:**
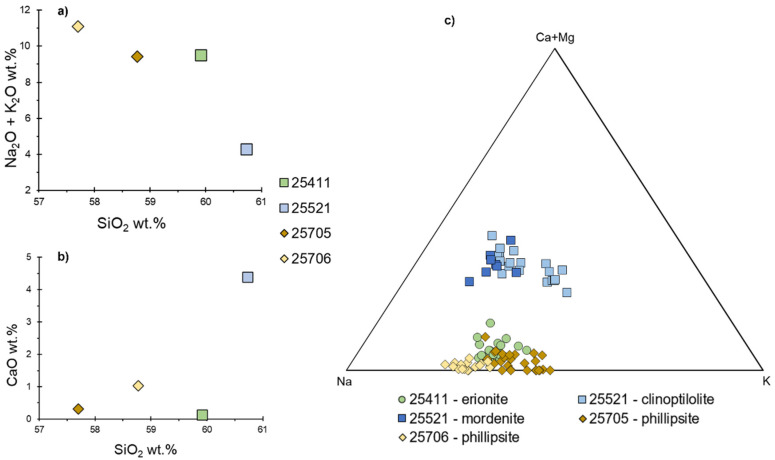
(**a**) Na_2_O + K_2_O vs. SiO_2_ (wt.%) and (**b**) CaO vs. SiO_2_ (wt.%) binary diagrams of the investigated tuff samples; (**c**) ternary diagram showing mole plot of exchangeable cation content in the zeolites detected within the tuff samples.

The chemical composition of the investigated tuff samples is difficult to differentiate, except based on their molar Si/Al ratio, varying from 6.29 to 10.24 wt.% (Table 2) and theirSiO_2_ values. As the Na_2_O + K_2_O vs. SiO_2_ and CaO vs. SiO_2_ binary diagrams of Figure 4a,b report, all the investigated tuff samples show a limited compositional range and the overall SiO_2_ values range from 57.7 to 60.7 wt.%. However, the 25411 and 25521 samples have slightly higher SiO_2_ values (59.9 and 60.7 wt.%, respectively) with respect to the 25705 and 25706 samples. Moreover, the 25521 sample has CaO values higher with respect to the other samples.

Crystallization of zeolite phases is the dominant process occurring within the analyzed samples. Thus, the following section focus on the dominant zeolites (erionite, mordenite, clinoptilolite and phillipsite) detected in the tuff samples. Representative electron microprobe analyses of the chemical composition are listed in Table 3. These data have been used to calculate the average chemical formulas of the zeolites, based on 72 oxygens for both erionite and clinoptilolite, on 96 oxygens for mordenite and on 32 oxygens for phillipsite. The chemical composition of the investigated zeolites appears reliable, as the sum of silicon and aluminum cations and extra-framework cations are stoichiometrically verified (see Table 4 for further details).

As shown by calculated chemical formulas, the dominant extra-framework cations of erionite [calculated formula: (Na_6.21_ K_3.33_Mg_0.25_Ca_0.20_) [Si_28.15_ Al_7.85_] O_72_ · 24.67 H_2_O] of the 25411 sample are Na and K, but also slightly lower Mg and Ca are present; in the clinoptilolite [calculated formula: (Na_2.49_ Ca_1.64_ K_1.57_ Mg_0.30_) [Si_30.20_ Al_5.80_] O_72_ · 20.94 H_2_O] of the 25521 sample Na and Ca are the dominant extra-framework cations, but also subordinate K and Mg are present; in the mordenite [calculated formula: (Na_3.44_ Ca_2.48_ K_1.83_Mg_0.25_) [Al_7.10_ Si_40.908_] O_96_ · 32.023 H_2_O] of the 25521 sample most of the extra-framework site is occupied by Na and Ca, with subordinate K and Mg; phillipsite [calculated formula: (Na_3.50_ K_2.37_ Ca_0.13_) [Si_12.95_ Al_3.05_ O_32_ · 11.99 H_2_O] of the 25705 sample have K and Na as dominant extra-framework cations, whereas in the phillipsite [calculated formula: (Na_4.28_ K_1.68_ Ca_0.04_) [Si_11.74_ Al_4.26_] O_32_ · 8.89 H_2_O] of the 25706 sample the dominant extra-framework cation is Na, with subordinate K. A ternary plot of the exchangeable cation content, displaying the above described features, is shown in Figure 4c.

The chemical compositions of the investigated tuff samples (Figure 4a,b) are consistent with the mineralogy and thus with the mole plot of exchangeable cation content (Figure 4c), in that the higher Na_2_O and K_2_O contents displayed by 25411, 25705 and 25706 samples and the higher CaO content displayed by the 25521 sample agree with their extra-framework cation population.

**Figure 5:**
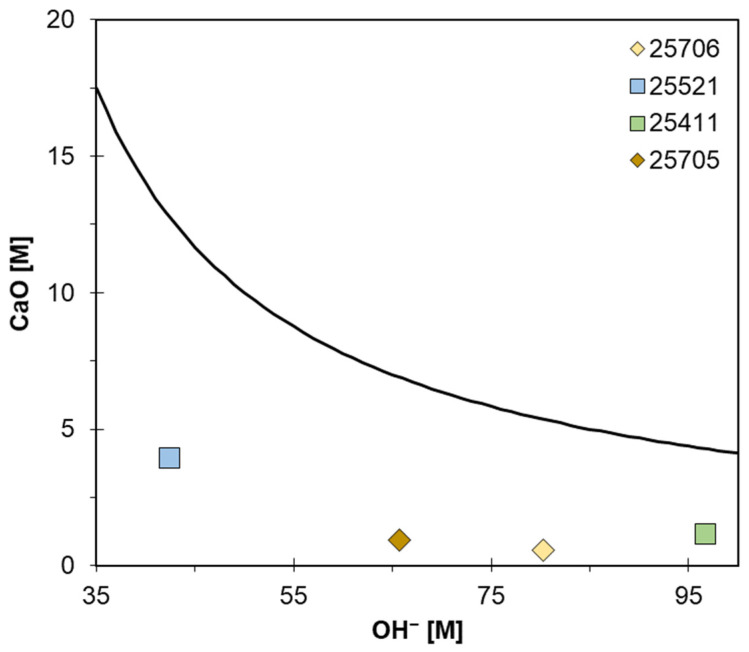
Results of Fratini’s test [51,52] for evaluating the pozzolanic activity of the investigated zeolites. Solid line represents Ca(OH)_2_ solubility curve.

Regarding the pozzolanic activity, Figure 5 shows the result of the test for estimating the reactivity of the various zeolite types detected in the analyzed tuffs as pozzolanic materials. All the investigated samples, having high zeolite content, provide under-saturation conditions, being positioned under the equilibrium Ca(OH)_2_ solubility curve. Zeolite addition caused a progressive decrease in Ca^2+^ and raising of OH- concentrations in solution.

These results allow us to quantify the pozzolanic activity of the investigated zeolites. Starting from the empirical formula [CaO_T_ = 350 ÷ (OH)^−^15], representing the theoretical solubility data of Ca(OH)_2_ (as CaO) at 40 °C in the 35–90 mmol l^−1^ [OH^−^] range, it is possible to evaluate the Ca(OH)_2_ reduction (in terms of CaO), and thus the effectiveness of the pozzolanic addition (E) [50]:

E = [(CaO_T_ − CaO) ÷ CaO_T_].

CaO_T_ is the theoretical concentration, calculated through the above empirical formula and CaO is the measured concentration obtained by Fratini’s test. The results of these calculations are reported in Table 4.

All the examined samples display a high reactivity with lime, with a Ca^2+^ reduction of at least 69% (Table 4). This means that zeolites (i.e., pozzolanic materials) react with calcium hydroxide, leading to the formation of insoluble calcium aluminate silicate hydrate (C-A-S-H) (acting as binding compounds). In fact, zeolites interacting with the solution modify its chemistry, decreasing Ca^2+^ concentration and increasing alkalinity (OH)^-^. Different simultaneous reactions and equilibria occur: (a) dissolution of solid Ca(OH)_2_ and related dissociation equilibria, (b) ion–exchange equilibria involving Ca^2+^ and Ca(OH)^+^ in solution and cation in zeolite and (c) breakdown and dissolution of zeolite in basic solution and/or conversion into a transient amorphous material, followed by the precipitation of hydrated calcium aluminates (CAH) and hydrated calcium silicates (CSH) [54].

The above results are also in good agreement with the theoretical Cation Exchange Capacity (CECteor) displayed by the investigated zeolites (Table 3). Moreover, despite all the zeolites are effective in removing Ca^2+^ from the solution, since all the experimental points are under the curve (Figure 4), 25706 sample appears to be more effective on controlling the Ca^2+^ concentration, with a Ca^2+^ reduction of about 90%. This tuff sample with the best pozzolanic behavior contains the less siliceous zeolites (i.e., phillipsite; cfr. Table 3) and this is probably the reason of its higher selectivity for Ca^2+^. In fact, phillipsites show a better capacity to exchange their extra-framework cation Na^+^ for Ca^2+^, as shown by the CEC > 3 with respect to the others (Table 3). At the same time, the 25705 and 25706 samples present a significant amount of amorphous phases, which further promotes their pozzolanic activity.

The presence of zeolites as additives in the blend can improve resistance of the cements to chemical attack, due to the decrease in CaO resulting from the hydrolysis of the hydrated calcium and aluminum silicates. Furthermore, the reduction of the alkali of the blend minimizes the risk of alkali–silica reactions, which can occur during cement setting between hydroxyl ions in the pore solution and the reactive components of the blend, leading to the formation of an alkali silicate gel and setting up the expansion forces that lead to the deterioration of the cement [55,56].

## 5. Conclusions

Results of the investigations carried out on erionite-, mordenite-, clinoptilolite- and phillipsite-bearing tuffs object of this work and the related obtained results allowed us to conclude the following:

(1) Detailed minero-petrographic and chemical characterization is fundamental for investigations about pozzolanic activity, for the definition of technological characteristics of geomaterials and for planning future developments and applications as well;
(2) All the investigated samples behave as good pozzolanic materials;
(3) All investigated zeolites are effective in reducing the concentration of calcium hydroxide in the solution in contact with the cement–zeolite blend;
(4) CEC values play a fundamental role in pozzolanic behavior: in fact, the less siliceous phillipsites of the 25706 sample behave as better pozzolanic materials due to their higher CEC with respect to the other zeolites;
(5) Given the good pozzolanic activity shown, all the investigated tuff samples can be advantageously used for pozzolanic cements preparation;
(6) Collected data aim at exploiting the technological value of zeolitic tuffs, natural resources other than pozzolana, in composite cement manufacturing.
(7) The use of mordenite- and erionite-bearing rocks analyzed here is controversial because, as well as asbestos, they have a fibrous habit and represent a risk for human health. In fact, if inhaled, they can cause, among other illnesses, lung damage, cancer and mesothelioma [56,57,58].

Future developments should regard the technical aspects related to the presence of such additives, by evaluating mechanical resistance of the hardened blended cements, containing zeolites with different characteristics, during the hydration reactions.

## Figures and Tables

**Table 1:** QXRPD results of the investigated tuff samples. reported in wt.%.

Sample	25411	25521	25705	25706
Amorphous content *	8	15	51	25
Quartz	2	3	3	1
Alkali feldspar	4	6	11	7
Plagioclase	4	3	7	-
Erionite	79	-	1	-
Mordenite	3	20	-	-
Clinoptilolite/Heulandite	-	52	-	-
Calcite	-	1	1	-
Phillipsite	-	-	25	65
Searlesite	-	-	1	-
Mica	-	-	-	2
Total	100	100	100	100

* By difference.

**Table 2:** Chemical analysis (XRF) of major oxides (wt.%) and LOI (wt.%) of the investigated tuff samples.

Sample	25411	25521	25705	25706
SiO_2_	59.91	60.73	58.77	57.70
TiO_2_	0.07	0.07	0.19	0.20
Al_2_O_3_	13.67	10.56	9.74	15.58
FeO	0.93	0.84	1.80	2.39
MnO	0.01	0.02	0.04	0.02
MgO	0.50	0.71	1.41	0.42
CaO	0.13	4.40	1.03	0.32
Na_2_O	5.81	2.01	4.20	7.54
K_2_O	3.70	2.28	5.22	3.57
P_2_O_5_	0.05	0.02	0.04	0.08
LOI	15.22	18.37	17.55	12.19
Si/Al	7.44	9.76	10.24	6.29

Notes: total Fe expressed as FeO; Si/Al = molar ratio.

**Table 3:** Chemical composition (wt.%) and CECteor (meq/g) of zeolites from the investigated tuff samples.

Sample	25411	25411	25411	25411	25411	25521	25521	25521	25521	25521	25521	25521	25521	25521	25521	25705	25705	25705	25705	25705	25706	25706	25706	25706	25706
	e	e	e	e	e	c	c	c	c	c	m	m	m	m	m	p	p	p	p	p	p	p	p	p	p
SiO_2_	58.35	58.32	63.38	59.72	60.3	67.36	65.56	70.05	68.61	69.89	69.16	67.72	71.1	66.59	68.03	56.6	54.28	55.94	56.11	58.86	56.52	58.34	56.14	59.52	60.46
TiO_2_	0.19	bdl	bdl	bdl	bdl	bdl	bdl	0.14	0.64	0.62	0.04	bdl	Bdl	bdl	bdl	bdl	0.38	0.08	0.2	0.19	bdl	0.24	bdl	0.08	0.05
Al_2_O_3_	13.61	14.33	16.03	13.38	14.32	11.52	9.99	11.85	11.54	10.11	10.92	11.99	10.33	10.56	8.98	14.08	14.82	13.94	12.46	9.24	17.5	18.54	18.02	18.73	17.28
FeO *	bdl	bdl	bdl	0.15	0.18	0.64	0.69	0.51	Bdl	0.82	0.09	0.43	0.98	bdl	0.37	0.23	0.3	0.23	0.58	bdl	0.72	0.17	0.29	bdl	0.72
MnO	bdl	bdl	bdl	bdl	0.31	0.07	0.01	0.09	0.1	0.16	0.31	bdl	0.35	bdl	bdl	bdl	0.1	0.03	0.07	0.15	bdl	bdl	bdl	0.15	bdl
MgO	0.27	0.36	0.4	0.38	0.03	0.11	0.24	0.08	0.31	bdl	0	0.46	0.34	0.31	bdl	bdl	0.36	0.24	0.07	bdl	0.01	bdl	0.23	bdl	0.05
CaO	0.22	0.14	0.47	bdl	0.13	2.51	2.39	3.32	2.08	2.65	2.42	2.43	2.85	2.34	2.54	0.04	0.38	0.6	0.25	0.26	0.17	0.09	0.01	0.19	bdl
Na_2_O	4.85	5.95	5.82	5.23	5.62	2.34	1.87	2.21	2.02	2	1.94	1.85	2.44	2.25	1.4	4.77	4.7	4.79	3.88	2.7	7.45	8.24	7.64	7.08	7.19
K_2_O	4.12	4.79	4.46	4.54	4.48	1.67	1.73	1.36	3.57	1.31	1.71	3.46	1.3	1.44	1.02	6.49	5.79	6.3	5.63	3.53	4.05	4.91	5.39	5.6	4.01
TOT	81.62	83.88	91	83.4	85.38	86.21	82.49	89.62	83.87	87.55	86.59	88.33	89.7	83.5	82.34	82.21	81.11	82.15	79.25	74.94	86.43	90.53	87.72	91.36	89.75
H_2_O	18.38	16.12	9	16.6	14.62	20.449	27.08	14.794	11.13	18.113	13.41	11.67	10.3	16.5	17.66	17.79	18.89	17.85	20.75	25.06	13.57	9.47	12.28	8.64	10.25
	cation based on 72 oxygens										cation based on 96 oxygens					cation based on 32 oxygens									
Si	28.191	27.721	27.659	28.334	28.05	29.966	30.411	29.942	29.836	30.499	40.599	39.574	40.56	40.479	41.607	12.313	11.992	12.218	12.574	13.491	11.700	11.583	11.548	11.683	11.962
Ti	0.07	-	-	-	-	-		0.045	0.208	0.202	0.019	0	-	-	-	-	0.063	0.013	0.033	0.034	-	0.036	-	0.011	0.007
Al	7.748	8.028	8.244	7.482	7.851	6.04	5.464	5.972	5.912	5.201	7.551	8.257	6.946	7.568	6.47	3.61	3.858	3.588	3.292	2.497	4.269	4.338	4.368	4.334	4.029
Fe	-	-	-	0.058	0.068	0.237	0.27	0.182	-	0.3	0.044	0.208	0.469	-	0.191	0.042	0.056	0.042	0.109	-	0.124	0.029	0.051	-	0.119
Mn	-	-	0.13	-	0.099	0.02	0.003	0.027	0.031	0.048	0.128	-	0.139	-	-	-	0.016	0.005	0.011	0.024	-	-	-	0.021	-
Mg	0.197	0.252	0.262	0.269	0.024	0.075	0.166	0.053	0.201	-	-	0.398	0.285	0.283	-	-	0.117	0.078	0.022	-	0.003	-	0.071	-	0.015
Ca	0.116	0.071	0.22	0.002	0.066	1.194	1.189	1.52	0.97	1.241	1.522	1.521	1.743	1.523	1.662	0.01	0.091	0.14	0.06	0.064	0.039	0.019	0.001	0.041	-
Na	4.545	5.48	4.92	4.814	5.072	2.015	1.685	1.832	1	1.689	2.205	2.1	2.703	2.655	1.663	2.014	2.015	2.03	1.684	1.202	2.991	3.171	3.045	2.695	2.759
K	2.538	2.904	2.485	2.746	2.658	0.947	1.024	0.742	1.981	0.727	1.283	2.579	0.945	1.113	0.795	1.8	1.63	1.755	1.609	1.031	1.069	1.243	1.414	1.404	1.011
H_2_O	29.61	25.554	13.099	26.263	22.671	20.449	27.08	14.794	16.141	18.113	26.242	22.729	19.593	33.449	36.019	12.903	13.914	13	15.501	19.153	9.364	6.268	8.421	5.658	6.758
R	0.784	0.775	0.77	0.791	0.781	0.832	0.848	0.834	0.829	0.854	0.843	0.827	0.854	0.842	0.865	0.773	0.757	0.773	0.793	0.844	0.733	0.728	0.726	0.729	0.748
Si/Al	3.638	3.453	3.355	3.787	3.573	4.961	5.566	5.014	4.858	5.864	5.376	4.793	5.84	5.349	6.431	3.411	3.108	3.406	3.82	5.403	15.969	15.921	15.915	16.017	15.991
Na/K	1.791	1.887	1.98	1.753	1.908	2.129	1.646	2.467	1.958	2.323	1.719	0.814	2.86	2.385	2.092	1.119	1.236	1.157	1.047	1.166	4.099	4.434	4.461	4.139	3.771
CECteor	2.66	3.16	3.19	2.84	2.83	2.058	1.94	2.23	1.97	1.87	1.85	2.43	2.25	2.02	1.57	2.93	3.06	3.212	2.57	1.71	3.33	3.73	3.73	3.54	3.20

Notes: bdl = below detection limits; * = total Fe expressed as FeO; H_2_O calculated by difference. m = mordenite; p = phillipsite; CEC values are expressed in meq/g; R = Si/(Si + Al); CECteor = theoretical Cation Exchange Capacity.

**Table 4:** OH and CaO concentrations and CaO_T_ and E evaluations obtained from the Fratini’s test.

Sample	OH^−^ mmol/L	[CaO] mmol/L	[CaO]_T_ mmol/L	E (%)
25706	80.23	0.55	5.37	89.8
25521	42.38	3.96	12.79	69.0
25411	96.65	1.14	4.29	73.3
25705	65.73	0.94	6.90	86.4

## Data Availability

All data derived from this research are presented in the enclosed figures and tables.
